# Magnetic-Measuring Square in the Measurement of the Circular Curve of Rail Transport Tracks

**DOI:** 10.3390/s20020560

**Published:** 2020-01-20

**Authors:** Arkadiusz Kampczyk

**Affiliations:** Department of Engineering Surveying and Civil Engineering, Faculty of Mining Surveying and Environmental Engineering, AGH University of Science and Technology, al. A. Mickiewicza 30, 30-059 Krakow, Poland; kampczyk@agh.edu.pl

**Keywords:** Magnetic-Measuring Square, MMS, radius of curve, railway track curve, curve versine, difference in rails length, rail shortenings, linear measurement procedure

## Abstract

In rail transport, measuring the actual condition of a circular curve of a railway track is a key element of track position monitoring not only during operation but also during final works. Predicting changes in its position in the horizontal plane is one of the most important related scientific issues. This paper presents the results of measurements performed with an innovative measuring device called the Magnetic-Measuring Square (MMS). The aim of the research was to demonstrate the acceptability of using the MMS. Horizontal versines of a rail track curve were measured as three neighboring points on a curve (using the method of lacing/stringlining, also called the three-point or the Hallade method), and the perpendicularity of rail joints and shortenings were measured. The MMS device presented in this article was used to measure versines and differences in rails lengths (rail shortenings in the curve) in the operating mode involving a laser distance meter with a laser beam (laser power P < 1 mW, laser wavelength λ = 635 nm) with a target cross, a camera, and a surveying measuring disk. The measurement results confirmed that it is possible to employ the MMS to monitor the geometry of railway track fragments such as track transition curves and railway track curves in rail transport.

## 1. Introduction

Three points on a plane determine a circle with a radius R, and the height of this triangle is the curve versine f_i_, while the base is the measurement chord c_i_. The shapes of track sections such as transition curves and curves are analyzed and evaluated on the basis of horizontal versine measurements. Adjustment of the track location in the horizontal plane by thrusting the track on curves and transitional curves should be verified through the measurement of versines. In reference to the topic of measuring the actual condition of a railway track curve, authors of numerous publications [1,2,3] have drawn attention to the fact that in order to minimize errors, land surveyors are obliged to take accurate measurements and properly select and calibrate measurement instruments depending on the purpose of the measurement [1]. Within the scope of preparatory works, the correct location of the track axis must be determined at the level of the track axis adjustment marks while thrusting the track. It has also been found out that if there are no adjustment marks near the track, then the displacement values are determined by using the values of versines measured every 5 m on a chord of 10 m [2]. Additionally, when the track is thrusted in its proper position, besides verifying the distance between track centers on multitrack railway lines, the preservation of the clearance gauge for structures and trolley poles, and the tilt, the values of versines on the transitional curve and the curve must also be ensured [2]. Transitional curves and railway track curves have different lengths in parallel matching, and the difference depends on the length and radius [4]. The value of the track radius (curvature) is determined by measuring the value of the versines. The state of the track curvature is assessed by dividing the curve into measuring sections of 5 m [3]. There are essentially, found that there are two methods for monitoring tracks. The first one assumes that the track is examined with optical instruments. This method is employed to analyze the geometrical quality of the track, and it is required for the designing processes related to comprehensive track works. In the second method, the railway track is examined from the chord to the running edge of the rail. The measured values are called versines, and measurements are carried out cyclically with overlapping strings. Attention has been drawn to the stringlining or the Hallade method [5]. The Hallade method was also referenced in [6,7,8,9,10]. Because curvilinear sections of the track are more prone to geometric irregularities than straight sections, they require regular maintenance and repair works [5]. The maintenance of the railway surface is its diagnostics together with the implementation of conclusions resulting from it, as well as planning and execution of maintenance and repair works [11]. A new system for correcting track irregularities has been developed using high-performance track machines (tamping machines). In this method, optimal data are calculated on the basis of measurements of track abnormalities using a three-point system [12,13]. The chord method is generally used for the horizontal and vertical versine measurements of rails in order to maintain their alignment [14]. In [14], the horizontal versines of the left and right rails were precisely calculated, and the vertical versine functions were exactly derived. According to these inferences, line charts for determining the vertical versine were created to demonstrate the proportional relationship with the grade difference and the curve length.

In turn, Wang et al. in [15] presented an error theory of the chord-based measurement system regarding track geometry and improvement by high-frequency sampling.

Yazawa and Takeshita [16] developed a device to measure track irregularity using an inertial mid-chord offset method. In [17], Zhang et al. stated that curve adjustment is an important part of railway maintenance and plays a major role in safe railway operations. To meet the requirements of calculating the versine in the lining and lifting work carried out by a tamping wagon, the authors of [17] proposed an approach for modeling the track curve and an algorithm for calculating the versine of any point in the rectangular coordinate system.

The issues involved in monitoring the state of railway track geometry were also dealt with by the authors of works [18,19,20,21] in reference to the use of railway vehicles. Seeing the same track repeatedly provides an opportunity to observe the degradation of track geometry, and such observations can potentially be used to inform maintenance decisions [18,19,20]. Maintaining the alignment of a railway track is vitally important for the smooth and safe passage of railway vehicles. Poor track alignment can result in poor ride quality, flange contact, or even flange climb [20]. In [21], the authors investigated the application of Doppler-based LIght Detection and Ranging (LIDAR) technology for determining track curvature and lateral irregularities, including alignment and gauge variation. The proposed method uses track measurements by two low-elevation, slightly tilted LIDAR sensors that are nominally pointed at the rail gauge face on each track. The study reported a close match between the LIDAR measurements and those made with existing sensors on-board the railcar [21]. Maciuk in [22] presented the advantages of combined GNSS (Global Navigation Satellite Systems) processing involving a limited number of visible satellites. The author noted that the accuracy level is needed to address certain surveying and civil engineering issues. In turn, in [23], the author observed that GNSS measurement techniques have recently acquired a major role in civil engineering and other technical fields. Examples of this application include the monitoring of both natural phenomena and fabricated constructions.

Al-Douri et al. in [24] confirmed that increased traffic has led to an increased rate of degradation of the railway track, which has resulted in higher maintenance costs. Degradation affects comfort, safety, and track quality, as well as reliability, availability, speed, and overall railway performance. By implementing a long-term maintenance strategy and conducting preventive maintenance actions, maintenance costs can be reduced [24]. In addition to that, problems with measured data, missing data, and incorrect location data have resulted in increased and unnecessary maintenance tasks. The conclusions in [24] show that proactive solutions are needed to reach the desired goals of improved safety, improved availability, and improved reliability. Chiacchiari and Loprencipe in [25] concluded that rail irregularities, particularly those in urban railway infrastructures, are one of the main causes of noise and vibration generation. Monitoring defects on a periodic basis enables a network’s rail managers to apply proactive measures to limit further damage.

**Figure 1:**
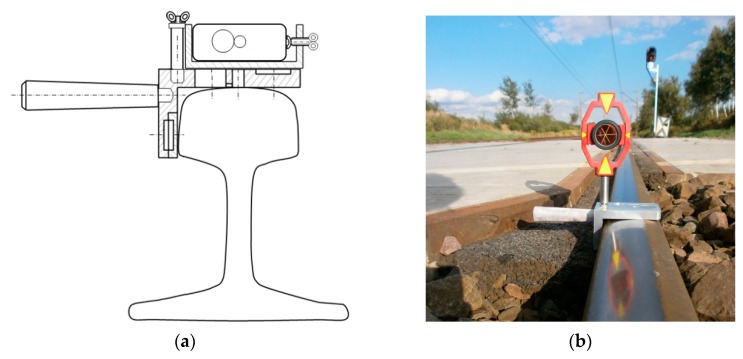

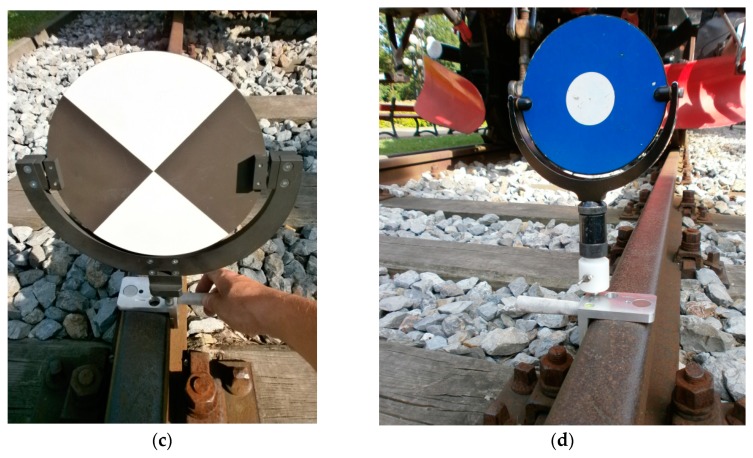
Application of the proprietary Magnetic-Measuring Square (MMS) in monitoring infrastructure of rail transport (own figure and photograph): (**a**) MMS in the cross section built on the rail head (crown of the rail); (**b**) MMS with Geodetic Mini Prism Type 111 (GMP111); (**c**) MMS with High-Definition Surveying (HDS) flat target Black & White and proprietary adapter made of insulating material for assembling reference signals to scanners; (**d**) MMS with HDS flat target Blue & White and proprietary adapter made of insulating material for assembling reference signals to scanners.

**Figure 2:**
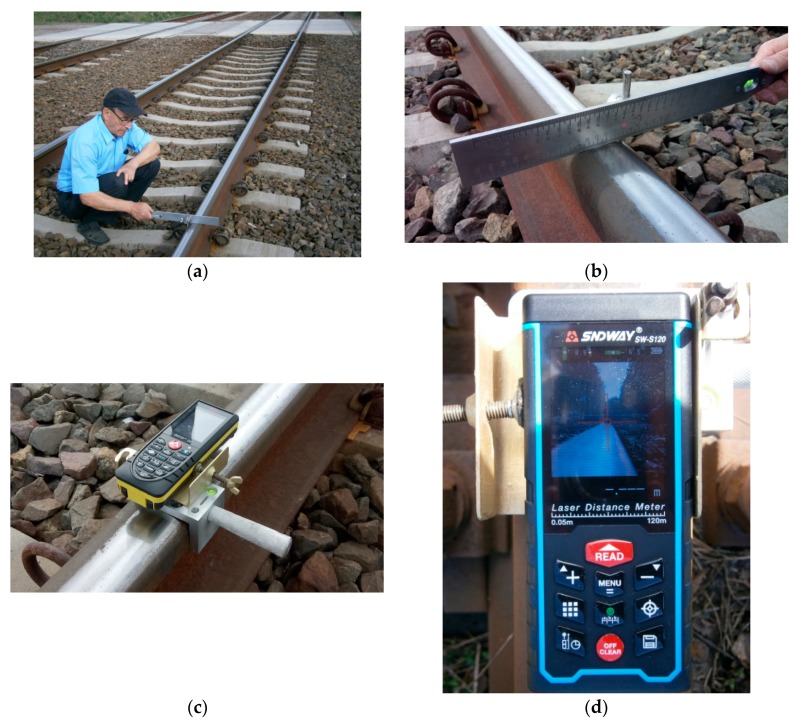
The Magnetic-Measuring Square for measuring versines (own photograph): (**a**) the lengths of the measurement base c_i_ = 10 m; (**b**) a measuring archet with a measuring archet adapter for vertical orientation; (**c**) a measuring square with a cap of a laser distance measuring device and the laser distance measuring device; (**d**) a measuring square with a laser distance measuring device with a visible pointing cross and video camera.

This paper presents results of measuring the real condition of a rail track curve and transitional curves with an innovative measuring device called the Magnetic-Measuring Square (MMS) [26]. Magnetic-Measuring Square is made of aluminium and metal. It is characterized by geometric features adapted to the structural elements in the rail transport infrastructure. The surfaces of the MMS are perpendicular to each other, in which neodymium magnets are installed and there are centering windows (centring over the point). The design of the measuring MMS ensures that the measuring work is carried out up to the lower edge of the rail head (crown of the rail) or at a height of 14 mm below the top running surface of the rail, depending on the measuring purpose [26]. The advantage of MMS is its compatibility inter alia with:

- Surveying pins prisms (adapter pin) for mounting various surveying prisms (Figure 1b);
- A surveying pin (adapter pin) with a guide for mounting a surveying target (disc) with an extended graduation;
- Reference signals for laser scanning (Figure 1c,d);
- Laser distance meter (LDM) with a laser beam (Figure 1a, Figure 2c,d and Figure 5a,b,d);
- A rolled up meter;
- Adapter for assembling reference signals to scanners (Figure 1d) [27];
- Specialised measuring strings;
- Horizontal and vertical measuring target (disk) (Figure 5a,c);
- Measuring archet (Figure 2a,b), measuring archet adapter.

The advantage of the Magnetic-Measuring Square is that it can be used universally and optimally for measurements. Finds a demand, among others in:

- Measurements of geometrical parameters of tracks and turnouts railways;
- Measurements of the horizontal and vertical versines;
- Checking the measurement of rails shortenings and perpendicularity of rail joint;
- Measurements of creeping of the rail (displacement continuous welded rail (CWR));
- The track zeroing measurements;
- Intertrack space measurements;
- Measuring the infrastructure gauge (structure gauge);
- Measurement of second differences (gradients) in the height of turnouts or diamond crossings;
- Levelling tracks and tornouts railways.

The aim of this research was to demonstrate the acceptability of using the MMS to measure the following:

- Versine measurements: the MMS was used in the operation mode with a taut measuring line (string) and laser distance meter (laser rangefinder) with a laser beam;
- Rail shortenings and the perpendicularity of rail joints: the MMS was applied in the operation mode with a laser distance meter with a laser beam and a horizontal surveying measuring disk.

The measurement was applied to track No.1 at the 14,860–15,760 km point, located within railway line No.144 Tarnowskie Góry–Opole Główne, Voivodship Silesian (in Polish: Śląskie), Poland. The characteristics of the geometrical layout of the track are as follows:

- km 14,860–14,980: entry transitional curve P_1_ = 120 m;
- km 14,980–15,640: full railway track curve R = 1090 m, L = 660 m;
- km 15,640–15,760: exit transitional curve P_2_ = 120 m.

The track grid, or so called track frame, is a connection between two rails by means of rail fastenings. The track grid together with the ballast forms a railway surface. Railway track surface is composed of UIC 60 type rails, sleepers, ballast and fixings, adapted to transfer loads of rail vehicles on the track bed. The sleepers are arranged perpendicularly to the axis of the track and their spacing is 0.70 m. The ballast is a layer of compacted gravel, laid under the sleepers and filling the space between them.

Reference was made to differences in the values of the versines on a measurement base of a 10 m chord as permissible deviations of the track location parameters, depending on the permitted speed on the track. There are also some principles for calculating and laying the shortened rails in the railway track curves, and the differences in the lengths of the internal and external rails were determined. The possible applications of the MMS for monitoring the state under off-road conditions are presented.

## 2. Methods of Measurement Using the MMS

### 2.1. Versine Measurement

The actual condition of a railway track curve with transitional curves was measured using an innovative measurement instrument—the Magnetic-Measuring Square—in two independent modes (methods) of work [26]:

- Two rail measuring squares (left and right) with a pin with a guide, an edge plate, a taut measuring line, and a measuring archet in a horizontal plane (chord base length c_i_ = 10 m);
- One measuring square with the cap of a laser distance measuring device, the cap installation pin, the stabilizer bracket, the laser distance measuring device (laser beam: class II; laser power P < 1 mW; laser wavelength λ = 635 nm), the edge plate, and the measuring archet, with an archet adapter providing vertical orientation (chord base length c_i_ = 10 m) (Figure 2).

In the first case, the versines were measured by using a chord with a taut measuring line, and in the second case, the versines were measured by using a chord with the laser beam.

**Figure 3:**
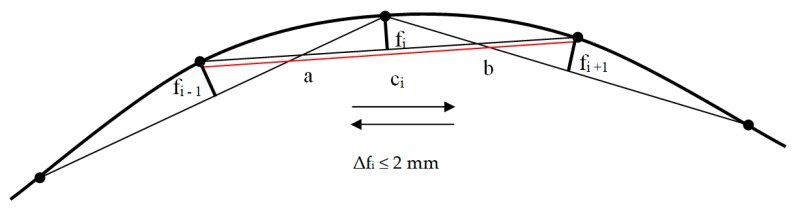
Horizontal versine measurement with the MMS (own Figure), where f_i_ is the horizontal versine of the curve, c_i_ is the chord base length, a and b are the distances between points, Δf_i_ is the difference between two measurements and f_i-1_ the value of the previous versine to the versine f_i_, f_i+1_ value of the next versine to the versine f_i_.

The track No.1 fragment at the 14,860–15,760 km point was divided into 5 m measurement sections and marked accordingly before the works started (abscissa x_i_ of the versine measurements). The measurement points were marked on the side surface of the rail head and neck (in a way that did not lead to their destruction by the traveling rolling stock). Two measurement sections form a single measurement base c_i_. The versine measurements were carried out according to the chord c_i_ = 10 m. Duplicate and independent measurements were taken for both the MMS with the taut measuring line and the MMS with the laser distance measuring device with a pointing cross and a video camera: the first measurement was in the main direction, and the second was in the return direction (Figure 3). Dependence between the versine f_i_ and the curve radius R was determined by Equation (1):

(1) $$f_{i}=\;\frac{a\;\cdot b}{2\;\cdot R}\;=\;\frac{a\;\cdot b}{2}\;k\;$$

where f_i_ is the horizontal versine of the curve, R is the curve radius, a and b are the distances between points, and k is the curvature of the track (inverse of the curve radius). At a given point in the curve, the curvature is the inverse of the radius. From the curvature and how it changes depends on train speed [28]. In a straight line the curvature at each point is equal to zero k = 0. On the other hand, on a circular arch, the curvature at each point of the arch is constant and equal to the inverse of the curve, determined by Equation (2):

(2) $$k=\;\frac{1}{R}\;$$

The state of track curvature characterizes the course of the measured versines. For easy orientation of the curvature of the track, the versines are measured at fixed intervals at half the length of the measuring chord.

### 2.2. Measurement of Rails Shortenings and the Perpendicularity of Rail Joints 

**Figure 4:**
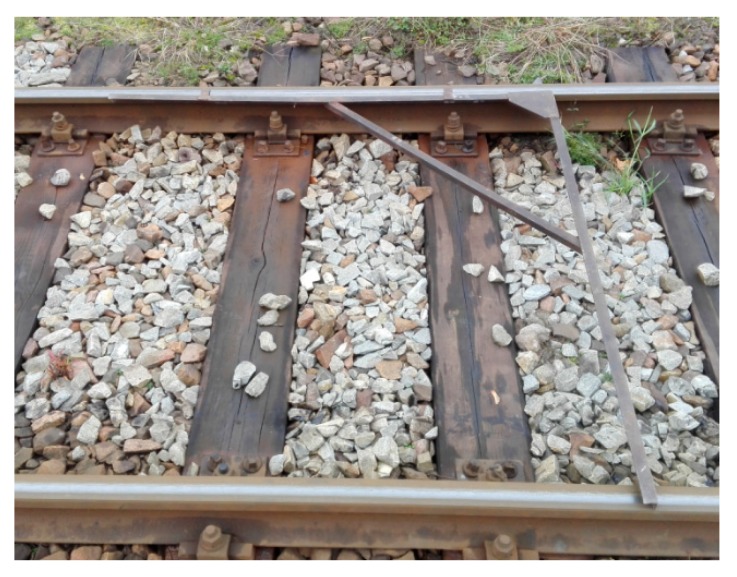
Track square (own photograph).

**Figure 5:**
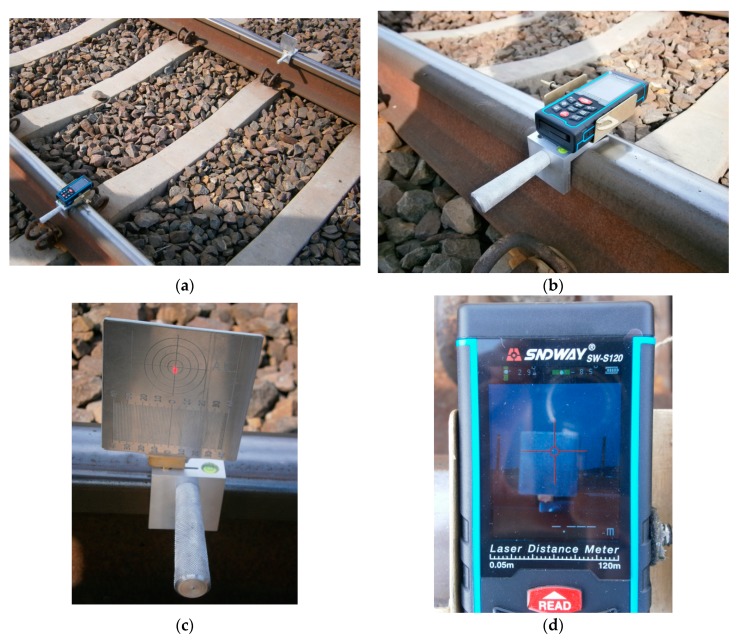
The Magnetic-Measuring Square (MMS) for measurements verifying the perpendicularity of the rail joints and shortening positions (own photograph): (**a**) the MMS location within rails; (**b**) the measuring square with a cap of the laser distance measuring device and with the laser distance measuring device; (**c**) the measuring square with a horizontal surveying measuring disk (with a millimeter scale with concentric rings); (**d**) the measuring square with a cap of the laser distance measuring device and with the laser distance measuring device with a visible surveying measuring disk, cross pointer, and video camera.

The adequate and correct position of the opposite joints has a certain impact on the smooth travel of a railway rolling stock. To date, the instrument employed to verify the perpendicularity of opposite rail joints and the position toward the track axis has been the track square, which is applied to the rail heads of the external and internal rails (Figure 4). The Magnetic-Measuring Square (Figure 5) is an innovative instrument that allows for the verification of:

- the perpendicularity of rail joints to the track axis,
- the perpendicularity of opposite rail joints, and
- rail shortenings.

For measurements of the state of the perpendicularity of rail joints and shortenings in the classical track, the MMS is equipped with the following [26]:

- A measuring square with a cap of a laser distance measuring device, cap installation pin, stabilizer bracket, and laser distance measuring device with or without an edge plate (Figure 5a,b,d);
- A measuring square with a horizontal surveying measuring disk (equipped with amillimeter scale and concentric rings) with or without an edge plate (Figure 5a,c,d) or with a surveying pin (adapter pin) with a guide for mounting a surveying target (disc) with an extended graduation.

The MMS structure is designed in such a way that the surveying measuring disk can be rotated by 180°, i.e., in the second position, and it has a vertical millimeter scale with concentric rings on the other side. It can then check the horizontality of the parallel rail joint locations and their underestimation (especially those of the traditional fishplate joints). The zero “0” value on the millimeter scale of the surveying measuring disk overlaps with the edge and measuring surface and with the cut of the pointing cross. The structure of the MMS allows it to take measurements to a height of 14 mm below the upper rail running surface or to the lower rail head edge (the most protruding point of the rail head). The MMS has been employed for the measurement of railway turnouts [29], the geometry of railway and road crossings, and the structure gauge [30,31], among other measurements.

External and internal rails fragments that correspond to each other and are contained within the same radius have different lengths. The inner rails is shorter than the external one. Therefore, the internal rail of the same cell should be shorter than its corresponding external rail. For railway track curves characterized by a high curve radius, the difference in lengths is small, and it can be leveled by clearance to some extent [32]. Maintaining the correct opposite position of the joints requires the use of leveling rails [32]. The leveling is carried out by placing:

- Regular rails within the external rails, and
- Shortened rails within the internal rails.

**Figure 6:**
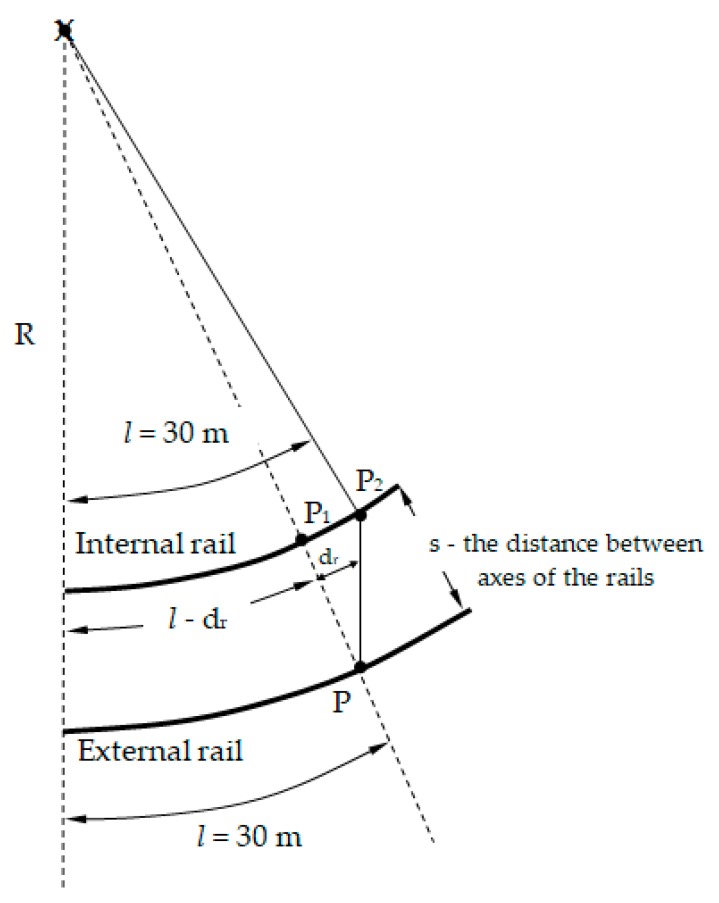
Measurement of the actual rail shortening (own Figure).

Corresponding themselves sections of the external and internal rail, contained between the same radii, have different lengths. The internal rail is shorter (Figure 6). In order to carry out measurements of rail shortening with the MMS device, the matching themselves sections of external and internal rails were divided into lengths l = 30 m. In the next stage, a projection of the obtained end point P on the inner rail was carried out, obtaining a point P_1_. The difference between the with projection point P_1_ on the inner rail and the end point P_2_ is the value of the real d_r_ rail shortening (Figure 6).

## 3. Results

### 3.1. Analysis and Evaluation of the Results of Versine Measurements Obtained from the MMS

**Figure 7:**
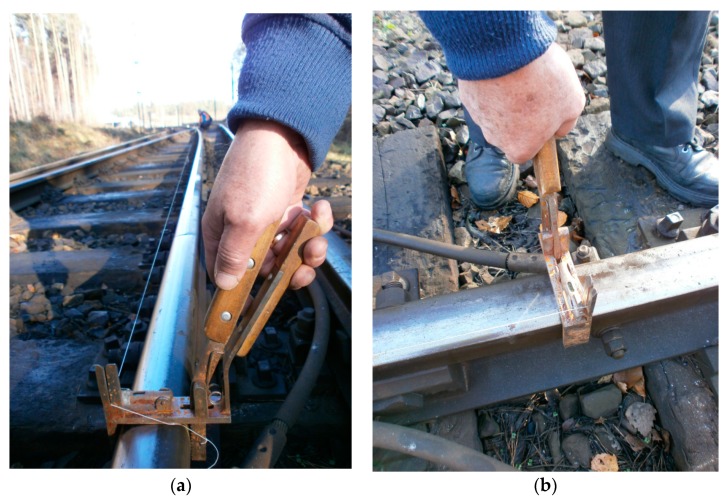
The Versine Measuring Device (VMD), as a regular device used so far) (own photograph): (**a**) the VMD—side view; (**b**) the VMD—overhead view.

Not depending on the measurements performed with the MMS, the measurement of the versines has also been made with an additional so-called Versine Measuring Device (VMD, as a regular device used so far) (Figure 7). The value of the average versine obtained with Versine Measuring Device f_spd_ ≈ 12 mm, actual radius of the railway track curve R_pd_ = 1040 m.

The VMD with metal handles is a device designed only for measuring arrows, used so far (Figure 7). It consists of two metal handles connected together by a string. The string, through the VMD construction, coincides with the lower edge of the crown of the rail. The metal handles are equipped with libellas. The VMD grippers have jaws to ensuring that it is applied to the lower edge of the crown of the rail. The gripper handle ensures that the jaw is placed on a rail and pressed against the edge. The measurement is done analogously according to Figure 3. The vesines measurement with the VMD can only be performed on days without wind. The VMD design makes it difficult to measure versines in turnout tracks.

**Figure 8:**
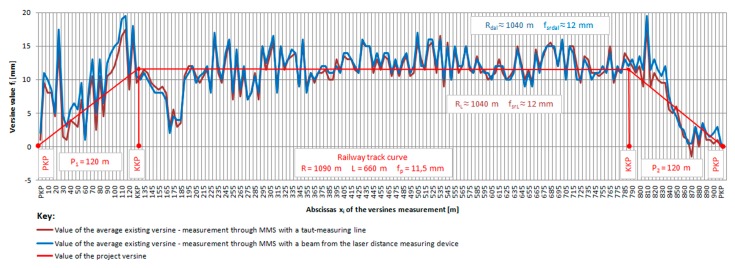
A diagram of horizontal versines in the exploited track (measurement on the basis of the measurement chord c_i_ = 10 m (own Figure).

**Figure 9:**
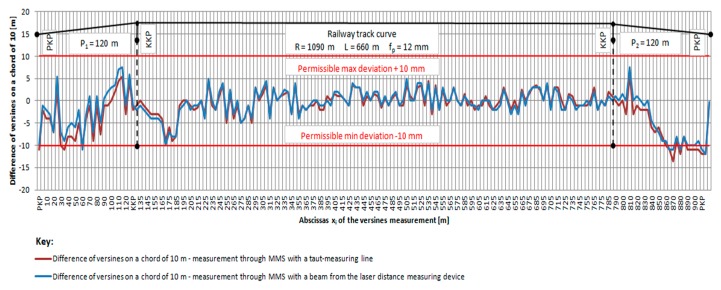
A diagram with differences of horizontal versines on the basis of the measurement chord c_i_ = 10 m (own Figure).

In each measurement method, the difference between the two measurements did not exceed the value of Δf_i_ ≤ 2 mm. Three neighboring points of the curve were considered during the measurements of versines in each measurement base c_i_ (Figure 3). The distance of the central point of the straight line connecting two other points (chords c_i_) is the versine f_i_ of the examined curvature. The measurements were considered satisfactory (Figure 8 and Figure 9). The difference between the value of the versine measured in the main and return direction (between the first and second measurement) must not exceed 2 mm [28]. Exceeding this value qualifies the measurement to be repeated [28]. For the MMS with the taut measuring line, the influence of wind was observable while taking the measurements, and this required the measurements to be repeated in order to maintain an accuracy of 2 mm. When it comes to measurements using the MMS with a laser distance measuring device (laser beam), the impact of weather conditions, especially sunlight, was observable. Satisfactory results could be obtained on cloudy days. The actual course of the track section condition was characterized by the values of the measured versines. The maximum difference between the MMS measurement with the taut measuring line and the MMS with the laser beam was 3 mm. The value of the design versine for a full curve with a radius R = 1090 m was f_p_ = 11.5 mm ≈ 12 mm. The actual value of the average versine obtained from the MMS measurement with the taut measuring line was f_srL_≈ 12 mm, and the value from the MMS with the laser distance measuring device was f_srdal_ ≈ 12 mm. The value of the actual railway track curve was R_L_ = R_dal_ = 1040 m.

The analysis and evaluation of the shape of the railway track section that includes the transitional curves and the railway track curve were carried out for a speed of V_max_ = 120 km/h. The value of permissible deviations from the basic parameters of the track locations within the difference of a versine value on a chord of 10 m for V_max_ = 120 km/h should not exceed ± 10 mm for exploited tracks [11]. This deviation for tracks in final commissioning upon maintenance, major repair, or modernization is the most restrictive. For a 10 m chord, the difference in the value of the actual versine measurement using the MMS with the taut measuring line f_srL_ and using the laser distance measuring device f_srdal_ was determined in relation to the design versine f_p_. The analysis proved that the differences in versine values were exceeded on the entry and exit transitional curve sections (Figure 9). There is a road and railway crossing zone on the section of the entry transitional curve P_1_ at measurements points 110–120. Ensuring the safe exploitation of the examined track section requires the introduction of a speed limit of V_o1_ = 100 km/h at the 14,860–15,640 km point on the entry transitional line P_1_. With this speed, the permissible difference in the versine value on the 10 m chord should not exceed ± 12 mm. For the exit transitional curve P_2_ at the 15,640–15,760 km point, it is recommended to introduce a speed limit of V_o2_ = 80 km/h. Within this speed, the permissible difference in the versine value on the 10 m chord should not exceed ± 14 mm. There is also a need to adjust the track axis in order to maintain the maximum speed.

### 3.2. Analysis and Evaluation of the Results of the Measurement of Rails Shortenings Obtained from the MMS

In a railway track curve with a radius R = 1090 m and a length L = 660 m (left direction toward increasing km points) and the length of each transitional curve P_1_ = P_2_ = 120 m, it is proposed to lay UIC 60 type rails with a length of L = 30 m and employ traditional shortening of rails d = 45 mm for L = 30 m. Laying the internal rails of the curve requires the use of standard length rails in addition to the shortened ones.

The number of shortened rails necessary for each curve should be determined by calculations. For a railway track curve that forms a full circle, the internal course of the rails is shorter than the external one by a value determined according to (3) [32]:

(3) $$d_{c}=2\pi \;\left(R\;+\;\frac{s}{2}\right)-\;2\pi \;\left(R-\;\frac{s}{2}\right)=2\pi \;\cdot s$$

where s is the distance between axes of the rails (1.5 m), and R is the curve radius in the track axis [m].

Given a distance between rails axes of 1.5 m, Equation (3) is transformed into Equation (4):

(4) $$d_{c}=2\pi \;\cdot s=2\;\cdot 1.5\pi =3\pi \;$$

In a railway track curve of length L, the difference in the length of both rails is expressed by Equation (5):

(5) $$d_{L}=3\pi \;\cdot \;\frac{L}{2\pi \;\cdot R}=\;\frac{3L}{2R}=\;\frac{3\;\cdot 660}{2\;\cdot 1090}=\;\frac{1980}{2180}=0.91\;m$$

Whether only the shortened rail or both shortened and regular rails should be laid within a particular curve is determined on the basis of the railway track curve radius, as well as the lengths of the regular and shortened rails. They are distributed along certain sections of the internal rails in so-called rail series. There is a need to calculate the curve radius R_min_, within which the internal course of only the shortened rails should be laid. For curves with radius values greater than the calculated R_min_, shortened and regular rails are laid in series. While determining the values for rail shortening, it must be borne in mind that for certain radius values, the internal course will be composed of only shortened rails [32].

Rails shortened by values that are multiples of 45 or 40 mm should be used in internal rails of traditional tracks. For new shortened rails, the rated shortenings 45, 90, 135, and 180 mm are applied [11]. The regular lengths of rails are 15, 18, 25, and 30 m. The curve radius R_min_ in which only shortened rails are laid is calculated using Equation (6) [33]:

(6) $$R_{\min}=\;\frac{1.5\;\cdot l}{d}=\;\frac{1.5\;\cdot 30}{0.045}=1000\;m\;$$

where R_min_ is the curve radius in which only shortened rails are laid [m], l is the regular length of rails [m], d is the assumed rail shortening [m], and 1.5 is the distance between axes of two rails [m].

According to the performed calculations, the radius of the curve in which only shortened rails are laid should be R_min_ = 1000 m. For curves with radius values larger than 1000 m, in addition to the rails shortened by 45 mm, regular rails with a length of l = 30 m should also be laid (rail cell of 30 m). The number of shortened rails that must be laid in the internal rails of the railway track curve N_1_ is (7) [33]:

(7) $$N_{1}=\;\frac{1.5\;\cdot L}{d\;\cdot R}=\;\frac{1.5\;\cdot 660}{0.045\;\cdot 1090}=\;\frac{990}{49.05}=20.2\;\approx 20$$

where N_1_ is the total number of shortened rails of a railway track curve, without consideration of transitional curves; L is the length of the curve [m]; 1.5 is the distance between axes of two rails [m]; R is the curve radius [m]; and d is the assumed rail shortening [m].

Twenty shortened rails were adopted. Shortening of the internal rails in relation to the external rails for the whole length or part of the curve is calculated according to Equation (8) [33]:

(8) $$D=\;\frac{1.5\;\cdot L}{R}=\;\frac{1.5\;\cdot 660}{1090}=0.91\;m$$

where D is the difference in length between the internal and external rails [m], L is the length of the curve [m], and R is the curve radius [m]. This value confirms the result of the calculations in Equation (5).

Next, the number of shortened rails N_2_ necessary for a given railway track curve considering the transitional curves is determined using Equation (9) [32]:

(9) $$N_{2}=\;\frac{1.5\;\cdot L_{1}}{d\;\cdot R}=\;\frac{1.5\;\cdot 780}{0.045\;\cdot 1090}=23.8\;\approx 25$$

where N_2_ is the total number of shortened rails of a railway track curve, with consideration of transitional curves; L_1_ is the length of the railway track curve that includes half the length of both transitional curves [m]; 1.5 is the distance between axes of two rails [m]; R is the curve radius [m]; and d is the assumed rail shortening [m].

The number of shortened rails and their distribution within a curve with a radius R = 1090 m with transitional curves that each have a length of 120 m and a railway track curve of length L = 660 m were determined by establishing the following:

- The calculated length is $L_{1}=660+\;\frac{2\;\cdot 120}{2}=780\;m$;
- The shortening of the internal rails is $\frac{3\;\cdot {\;L}_{1}}{2\;\cdot R}\;=\frac{3\;\cdot \;780}{2\;\cdot 1090}=1.073\;m$;
- The assumed rail shortening is 45 mm;
- The whole calculated length requires 1.073/0.045 = 23.8 ≈ 25 shortened rails;
- The assumed length of a regular rail is l = 30 m;
- The relation of shortened to regular rails is $\frac{25}{30}=\;\frac{5}{6}$.

Therefore, for every six regular rails in the external rails, the internal rails has five shortened rails and one regular rail. The shortened rails should be laid relatively symmetrically about the center of the curve. However, if the curve is long, they should be laid in the same groups so that the deviations of the internal rails joints from the common radius are as small as possible. At the same time, the shortened and regular rails should be distributed as evenly as possible in each series. A series is composed of six rails. The number of series is $\frac{780}{6\;\times \;30}=4.3$.

In the examined track No.1, measurements of the actual state rails shortenings were carried out. The analysis and evaluation of the obtained (real) values of the rails shortenings was carried out in relation to the design values. The results of actual rails shortenings for selected series were as follows:

- Seria A: 42; 45; 45; 46; 47 mm;
- Seria B: 44; 46; 46; 45; 44 mm.

The average value of the obtained real rails shortenings in the analyzed series A and B was 45 mm. The design rails shortenings adopted in accordance with recommendations of Id-1 (D-1) [11] is 45 mm. The minimum difference between the average value of a rails shortenings and the designed one is 0 mm and the maximum value of the difference is 3 mm. Rail contacts in curved track shall be in line with the curve radius. The permissible deviation from this rule may not exceed half of the shortened value of a single rail according to the recommendations of Id-1 (D-1) [11,34]. In the examined track No. 1 this condition has been preserved.

## 4. Discussion

The research conducted by the author focused on measuring the real state of a circular arch with transition curves using an innovative measuring instrument called the Magnetic-Measuring Square. The analysis of measuring instruments used to measure the actual condition of curvilinear sections, with the use of both measuring vehicles [18,19,20,21] and portable measuring equipment [6,7,8,9,10], has shown that monitoring the condition of a curvilinear section’s geometry is an important part of railway infrastructure maintenance and plays an important role in the safe operation of railways. In [8,35] they correctly state that measurements of track irregularities are based on the assumption that the curvature of the track can be determined by versines. Increased railway traffic leads to the rapid degradation of railway tracks [24], which significantly affects quality, comfort, and safety. The state of a curvilinear section’s (the actual circular curve condition with transition curves) geometry is particularly important, especially for tracks in operation, and requires the use of universal and optimal measuring instruments: the solution of this problem is the MMS.

The measurement of versine in curves is performed on the basis of 10 m chord, such recommendation results from both the Id-14 (D-75) [36] instruction and the Id-1 (D-1) [11] technical conditions. Additionally, Id-14 (D-75) [36] and Id-1 (D-1) [11] define the values of permissible basic deviations as the differences of versine on the chord measured 10 [m] (measuring base). The tests of track No.1 using MMS and Versine Measuring Device were carried out according to the requirements of these regulations. The distance between measured points is 5 m. According to the recommendations of Id-14 (D-75) [36] and Id-1 (D-1) [11] regardless of the value of the curve radius, the length of the chord is always 10 m and the distance between the measured points is 5 m.

However, the binding technical standard GK-1 [28] allows for the selection of the distance between the measured points depending on the value of the curve radius:

- 5 m for radius R < 250 m;
- 10 m for radius from R = 250 m to R < 800 m;
- 20 m for radius from R = 800 m to R < 2000 m;
- 25 m for radius from R = 2000 m to R < 4000 m;
- 50 m for radius R ≥ 4000 m.

The design value of the radius of the arc under examination is R = 1090 m, so the distance between the measured points 20 m, i.e. chord length 40 m, had to be taken. This recommendation is contrary to the requirements of Id-14 (D-75) [36] and Id-1 (D-1) [11], especially since they determine the permissible deviations of the versine values. By analysing and evaluating the state of curvature, they determine the appropriate speed in the track. These deviations depend on the speed in the track. In addition, using such a large value of the measuring chord (40 m), it is exposed to many errors and not really reflecting the state of curvature.

The author’s measurement and control system using the MMS was successfully applied for rail transport infrastructure. The measurement results show that the system is accurate, fast, safe, and reliable. It includes optimization through the use of the best (optimal) solution for the specific application. The versatility of MMS measurement allows for the acquisition of measurement data from active rail infrastructure. Special attention was paid to measurements taken in the laser rangefinder and laser beam modes, which, in contrast to the measurement line mode, are not susceptible to wind effects. An important design feature of the MMS is the ability to take measurements to a height of 14 mm below the upper rolling surface of the rail or to the lower edge of the rails.

The acquisition of observational data using laser beam solutions is able to complement or even replace traditional methods of obtaining geometric data of the real state of curvilinear track sections.

The MMS with the laser rangefinder with a laser beam with a visible target cross and camera was also used to monitor railway infrastructure elements, such as the structure gauge (infrastructure gauge), turnout track geometry, and diamond crossing, and take measurements for surveying the Railway Special Grid.

In the field of engineering and industrial geodesy, laser beams have been used for innovative solutions involving measuring instruments [37,38]. In [37], an autonomous measuring system of lifts’ long guide rail deformations was applied in the context of telemonitoring as a challenge for geodesy of the XXI century. In [38], a laser beam was used in a leveling measurement system using the LSS.

Measuring the actual state of a circular arch using the Magnetic-Measuring Square significantly shortens the time of periodical measurements, which is very significant for tracks in operation. The final measurement results of the actual state of a circular curve using the MMS are used to maintain the geometry of the exploited track, including the periodic adjustment of the track axis.

## 5. Conclusions

Satisfactory results were obtained by applying the Magnetic-Measuring Square to measure the actual condition of a railway track curve with transitional curves using two independent operating modes: one used a taut measuring line and the other used a laser distance measuring device equipped with a pointing cross and video camera. For measurements obtained by using the MMS with the taut measuring line, the impact of wind was observable. On the other hand, measurements carried out with the MMS with the laser beam of the laser distance measuring device and video camera depended on the influence exerted by strong insolation. The measuring instrument was subjected to direct tests in the field conditions of an operated railway line, enabling its operation with a single beam or laser plane.

The new measuring instrument was used and verified in terms of its construction by taking measurements on a 10 m chord. The laser rangefinder with a laser beam used in the MMS, with the selection of the appropriate power and wavelength, ensured that the measurements were carried out.

Within straight fragments of the track, the rail joint in both rails should be located within the same line, which is perpendicular to the track axis and within the same radius in the case of curves. According to [11], deviations from these principles must not exceed 20 mm for a straight track or half of the shortening value of a single rail for a track in a curve. In each series, the same number of regular rails should be accompanied by a constant number of shortened rails so that the joints of both rails at the beginning and at the end of each series lay as close as possible toward the radius [32]. On the basis of the performed examinations, it is recommended that the series of rails be distributed within the internal rails with an accuracy not greater than half of the rail shortening (as the deviation of joints in both rails from the opposite position).

## 6. Patents

Magnetic-Measuring Square and its application. Patent application No.P.420214. (Przykładnica magnetyczno-pomiarowa i jej zastosowanie. Zgłoszenie patentowe P.420214).

Bulletin of the Patent Office, 16, pp. 24, Warsaw.

Biuletyn Urzędu Patentowego, 16, pp. 24, Warszawa.

Adapter for assembling reference signals to scanners. Utility model PL 69951 Y1 (Adapter do montażu sygnałów referencyjnych do skanerów. Wzór użytkowy PL 69951 Y1).

Bulletin of the Patent Office, 17, pp. 41, Warsaw.

Biuletyn Urzędu Patentowego, 17, pp. 41, Warszawa.
